# Global research trends in biomarkers, therapeutic targets, and drugs for amyotrophic lateral sclerosis: a bibliometric and visualization analysis

**DOI:** 10.3389/fphar.2025.1588968

**Published:** 2025-04-07

**Authors:** Na Guo, Weicheng Huang, Jianliang Huang, Ying Liu, Kai Zhu, Wei Gao

**Affiliations:** ^1^ Center for Experimental Medicine, The Third Xiangya Hospital, Central South University, Changsha, Hunan, China; ^2^ Department of Neurosurgery, Xiangya Hospital, Central South University, Changsha, China; ^3^ The Affiliated Cancer Hospital of Xiangya School of Medicine, Central South University/Hunan Cancer Hospital, Changsha, China; ^4^ The Key Laboratory of Carcinogenesis of the Chinese Ministry of Health, The Key Laboratory of Carcinogenesis and Cancer Invasion of the Chinese Ministry of Education, Cancer Research Institute, School of Basic Medicine, Central South University, Changsha, China; ^5^ Medical College of Jishou University, Jishou, Hunan, China

**Keywords:** amyotrophic lateral sclerosis (ALS), bibliometric analysis, translational therapeutics, neurodegenerative biomarkers, interdisciplinary neurology, stem cell therapy, blood-brain barrier penetration, skeletal muscle targeting

## Abstract

**Background:**

Amyotrophic Lateral Sclerosis (ALS) is a fatal neurodegenerative disease characterized by progressive degeneration of motor neurons, marked by complex pathological mechanisms and a lack of effective treatments. Despite substantial global research efforts, no comprehensive bibliometric analysis has systematically mapped the evolution of ALS biomarkers, therapeutic targets, and pharmacological advancements.

**Methods:**

This study, based on 4,250 publications retrieved from the Web of Science Core Collection (2005–2025), employs bibliometric tools such as CiteSpace and VOSviewer to conduct the first multidimensional analysis of global trends in ALS biomarkers, therapeutic targets, and drug research.

**Results:**

The results revealed contributions from 20,168 authors across 92 countries, with annual publications growing at an average rate of 16.5%. The United States dominated research output, accounting for 34.07% (n=1,448, TLCS=7,100), while the United Kingdom achieved the highest research impact with an average of 68 citations per article. Leading institutions, including the University of Oxford and the University of Milan, consistently produced high-impact studies. Pioneering scholars such as Turner MR and Kiernan MC made significant contributions to advancing therapeutic targets and drug discovery. The interdisciplinary integration of molecular biology and genetics emerged as a core driver of progress in ALS research. Neurofilament light chain (NfL), antisense oligonucleotide (ASO) drugs, transcranial magnetic stimulation (TMS), oxygen free radicals (oxidative stress), and gene therapy have consistently remained central research focuses in the ALS therapeutic field. Looking ahead, stem cell therapy, blood-brain barrier (BBB) penetration technologies, and skeletal muscle targeting are poised to emerge as prominent research directions.

**Conclusion:**

The United States dominates ALS research productivity, whereas the United Kingdom demonstrates superior citation influence. Despite China’s substantial publication volume, its limited citation impact underscores the necessity for enhanced methodological rigor and strategic international collaboration. Current research priorities encompass NfL, TMS, and ASO therapies, with emerging innovations in stem cell therapy, BBB penetration technologies and skeletal muscle targeting showing therapeutic promise. Future directions should prioritize biomarker standardization, optimization of drug delivery systems, and Clinical Translation.

## 1 Introduction

Amyotrophic lateral sclerosis (ALS), a fatal neurodegenerative disorder marked by the progressive degeneration of motor neurons, continues to be one of the most challenging diseases to manage, with patients typically surviving only 2–5 years following diagnosis (Chio et al., 2013). Despite substantial progress in elucidating its pathophysiology, therapeutic options remain severely limited. To date, only two drugs, riluzole and edaravone, have received regulatory approval, providing modest survival benefits of a few months (Bensimon et al., 1994; Petrov et al., 2017). Riluzole, a glutamate antagonist, and edaravone, an antioxidant, target specific aspects of ALS pathology but have not significantly halted disease progression. This therapeutic gap highlights the urgent need for innovative approaches to address the multifaceted nature of ALS. The complex pathogenesis of the disease involves genetic mutations (SOD1, C9orf72), protein misfolding (TDP-43 aggregates), mitochondrial dysfunction, and neuroinflammation, all of which collectively contribute to motor neuron degeneration (Taylor et al., 2016). These diverse mechanisms underscore the critical need for a more comprehensive understanding of ALS biology and the development of targeted therapies that address its underlying causes, rather than merely alleviating symptoms.

Recent advancements in multi-omics technologies have significantly transformed the identification of potential biomarkers and therapeutic targets for ALS. Notably, neurofilament light chain (NfL) levels in cerebrospinal fluid and blood have emerged as promising indicators for early diagnosis and monitoring disease progression. Elevated NfL levels exhibit a robust correlation with motor neuron degeneration (Gendron et al., 2017). Moreover, extracellular vesicle-based biomarkers, which serve as indicators of neuronal and glial cell health, are increasingly recognized for their potential to offer non-invasive diagnostic and prognostic insights (Thompson et al., 2018). On the therapeutic front, genetic modifiers have emerged as a focal point, with antisense oligonucleotides (ASOs) targeting SOD1 and C9orf72 mutations demonstrating promising results in both preclinical studies and early clinical trials (Miller et al., 2020; Miller T. M. et al., 2022). In addition to genetic approaches, strategies that focus on neuroprotection are being actively explored. These strategies include modulating glutamate excitotoxicity and oxidative stress, as well as addressing glial cell dysfunction (Paganoni et al., 2020). These advancements highlight the rapid progression and dynamic evolution of ALS research, providing hope for the development of more effective treatments that can tackle the disease’s complexity and heterogeneity.

Bibliometric analysis, a quantitative method for mapping research trends and collaborations, has emerged as an indispensable tool in synthesizing fragmented knowledge and identifying translational opportunities (Huang et al., 2024; Ninkov et al., 2022; Wilson et al., 2021; Liu et al., 2025). By systematically analyzing data from extensive literature, it uncovers developmental trends and research hotspots in science. In amyotrophic lateral sclerosis, bibliometric analysis can elucidate advances in therapeutic targets and drug development, thereby enhancing our understanding of disease mechanisms, guiding the development of new therapies, and improving diagnostic approaches. However, prior studies have largely examined ALS pathophysiology or drug development in isolation, without integrating biomarkers, therapeutic targets, and drug discovery within a unified bibliometric framework. To address this gap, we conducted a comprehensive bibliometric analysis of global research trends in ALS biomarkers, therapeutic targets, and drug development from 2005 to 2025. Using co-citation analysis, keyword clustering, and author-institution analysis, our study not only fills existing gaps in the bibliometric but also systematically evaluates current therapeutic strategies. By clarifying research priorities and hotspots in this field, we provide rational projections for future directions.

## 2 Materials and methods

### 2.1 Data sources and search strategy

**FIGURE 1:**
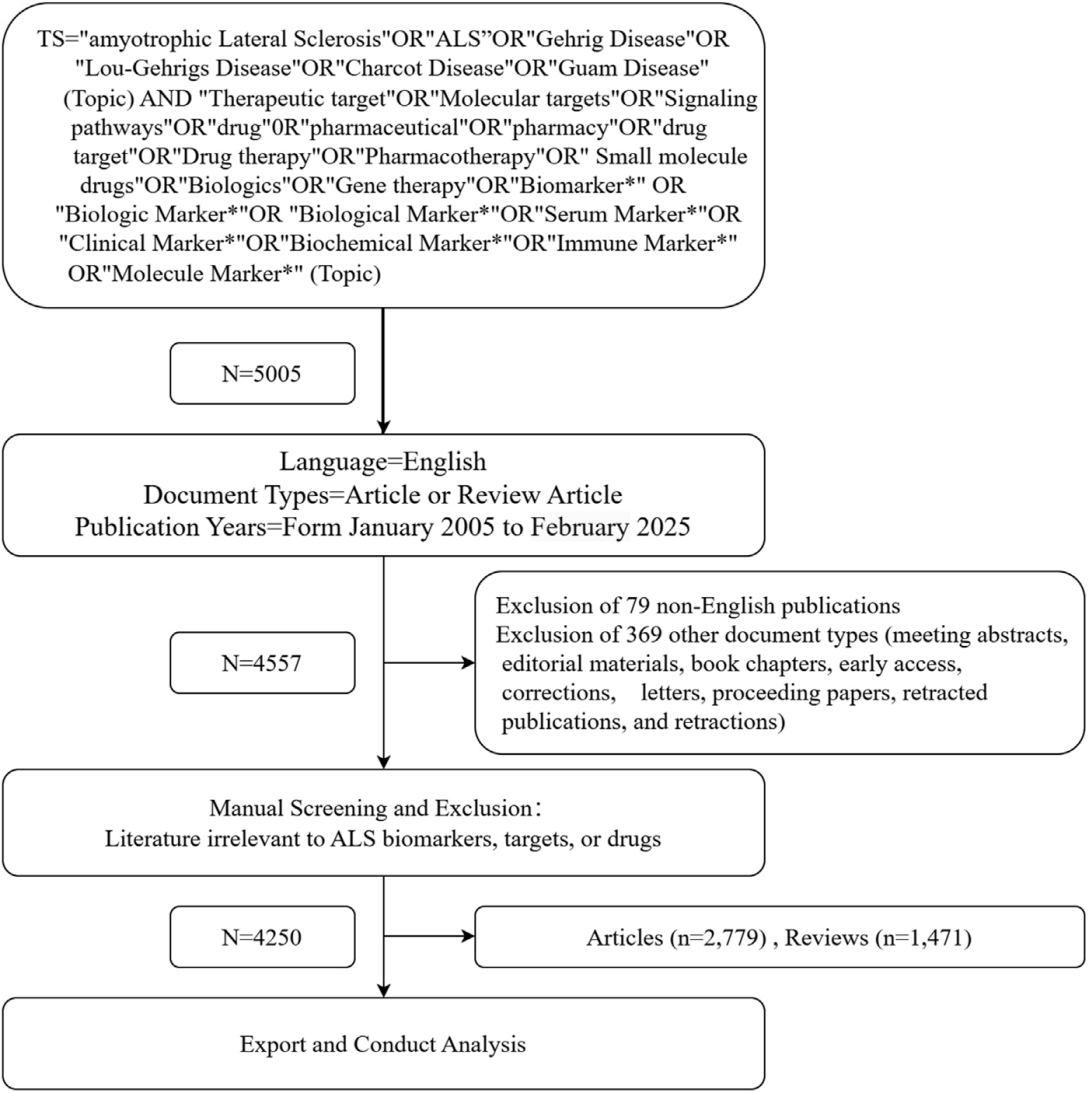
Flow chart of data collection in this study.

This study retrieved literature related to ALS biomarkers, therapeutic targets, and drugs from the Web of Science Core Collection (WOSCC) database over the past 20 years (January 2005–February 2025). To ensure data accuracy, all searches were completed on 16 February 2025. Figure 1 illustrates the data retrieval and inclusion process. The search strategy combined the following terms under the “Topic” field: amyotrophic lateral sclerosis, ALS, Gehrig Disease, Lou-Gehrigs Disease, Charcot Disease, and Guam Disease, which were linked using the Boolean operator “OR.” These terms were further combined with “AND” to include keywords such as Therapeutic target, Molecular targets, Signaling pathways, drug, pharmaceutical, pharmacy, drug target, Drug therapy, Pharmacotherapy, Small molecule drugs, Biologics, Gene therapy, Biomarker*, Biologic Marker*, Biological Marker*, Serum Marker*, Clinical Marker*, Biochemical Marker*, Immune Marker*, and Molecule Marker*. Filters were applied to limit publications to the years 2005–2025, document types to articles or review articles, and languages to English. Manual screening was conducted to exclude studies unrelated to the research focus. Finally, comprehensive bibliographic data, including titles, authors, affiliations, keywords, publication years, and references, were exported for subsequent analysis.

### 2.2 Research methodology

In this study, we integrated multi-platform software tools, including HistCite Pro 2.1, VOSviewer 1.6.19, Pajek 1.0, Microsoft Excel, and CiteSpace 6.1.6, to perform bibliometric data processing and visualization analysis. CiteSpace 6.1.6, a science mapping tool for bibliometric analysis, employs burst detection to identify rapidly emerging keywords and analyzes co-citation relationships among publications and journals to generate cluster labels and timeline views (Chen et al., 2012). This software was pivotal in tracking the evolution of research hotspots, identifying key transformative studies, and mapping trends in ALS biomarkers, therapeutic targets, and drug development. This study implemented scientometric analysis and visualization using CiteSpace software based on Kleinberg’s stream data state transition model. The analytical framework was configured as follows: Time slicing was first segmented into annual intervals. For constructing co-citation networks and keyword co-occurrence networks, cosine similarity was applied to calculate node linkage strength, with network boundaries constrained within temporal slices. The burst detection module employed a two-state Markov model, where the state transition threshold was set to two standard deviations and the minimum burst duration was defined as 2 years. The Global Citation Score (GCS) refers to the total number of times a publication has been cited across the global academic database, reflecting its broad influence within the entire scholarly community. In contrast, the Local Citation Score (LCS) measures the citation count of a publication within a specific research domain, highlighting its significance in the context of ALS biomarkers, therapeutic targets, and drug development. HistCite Pro 2.1 identifies high-impact countries, leading institutions, prominent authors, key journals, and core publications within the field by calculating the Global Citation Score (GCS) and Local Citation Score (LCS) of scholarly works, thereby supplementing and validating the findings derived from CiteSpace. VOSviewer 1.6.19 employs density and distance metrics to generate visualization maps, including network visualization and overlay visualization, which intuitively illustrate evolving dynamics within research communities and shifts in topic prominence. Collectively, these tools transformed vast literature into structured knowledge, revealing the evolutionary dynamics and core research forces in the field, while guiding future research directions.

## 3 Results

### 3.1 Annual publication trends

This study incorporated 2,779 articles and 1,471 reviews published between January 2005 and February 2025, totaling 4,250 publications. Within this timeframe, the earliest review in the field, titled “Issues for Clinical Drug Development in Neurodegenerative Diseases” by Dib,M et al. (2005) in Drugs, identified riluzole as demonstrating significant survival benefits in ALS clinical trials and highlighted the potential of cerebrospinal fluid glutamate concentration as a biomarker for tracking ALS progression.

**FIGURE 2:**
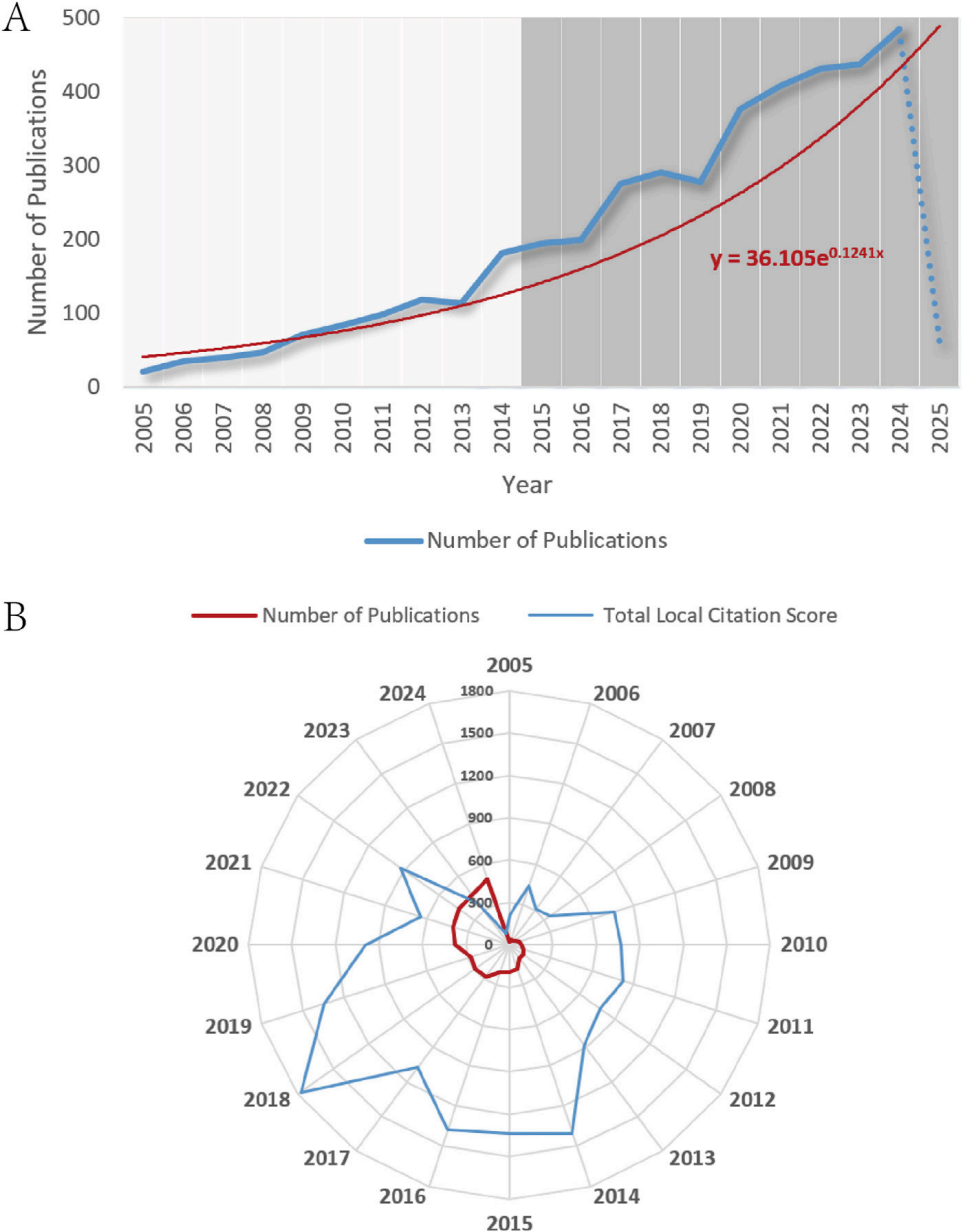
**(A)** Global annual output trends. **(B)** TLCS and growth rate in annual publications.

As illustrated in Figure 2A, annual publications exhibited a pronounced fluctuating upward trend, increasing from 21 in 2005 to 486 in 2024, representing a 22-fold growth with an average annual growth rate of 16.5%. The publication trajectory was divided into two distinct phases. The slow growth phase (2005–2013) saw publications rise from 21 to 114, with an average annual growth rate of 17.98%. As illustrated in Figure 2B, the Total Local Citation Score (TLCS) surged from 213 to 881 during the foundational research period (2005–2013), marking a 314% increase, while the average TLCS per article remained stable (7.4–12.7)0, indicative of high-quality studies in the field’s nascent phase. However, post-2014 saw a transition to a rapid growth period (2014–2024), with annual publications escalating from 182 to 486 (average annual growth rate: 19.8%). Concurrently, the average TLCS per article fluctuated between 4.6 and 7.7 from 2014 to 2019, reflecting a “dilution effect” characterized by reduced quality growth despite expanded output, as research scaled and diversified. Post-2019, TLCS continued to decline, likely attributable to lagging citation accumulation cycles. The significant increase in annual publication rates after 2014 may be attributed to heightened global attention resulting from the 2014 *Ice Bucket Challenge*—a large-scale international public campaign initiated to support individuals with amyotrophic lateral sclerosis (ALS). According to statistics, this campaign involved over 17 million participants (Sohn, 2017). And the 2015 approval of edaravone in Japan, which further galvanized research efforts. A polynomial fitting model (Y = 36.105e^0.1241x^) projected sustained rapid growth in publication volume, underscoring the field’s expanding momentum.

### 3.2 Country-level analysis

A total of 92 countries contributed to ALS-related research. Table 1 summarizes the publication volume, percentage of total output, TLCS, TGCS, and average citation rates for the top five countries. The United States dominated in scale, accounting for 34.07% of publications (1,448) and achieving a TLCS of 7,100. However, its average citation rate (53) lagged behind the United Kingdom (68), suggesting room for improvement in research quality. China ranked second in publication volume (553 articles, 13.01%) but had the lowest TLCS (747) and average citation rate (23). Despite ranking fourth in output (492 articles), the United Kingdom achieved the highest average citation rate.

**TABLE 1:** Top five countries with the most publications.

Lank	Country	Documents	Percent (%)	TLCS	TGCS	Average article citation
1	United States	1,448	34.07%	7,100	76,550	53
2	China	553	13.01%	747	12,751	23
3	Italy	539	12.68%	2,940	23,968	44
4	United Kingdom	492	11.58%	4,792	33,326	68
5	Germany	384	9.04%	2,973	19,396	51

**FIGURE 3:**
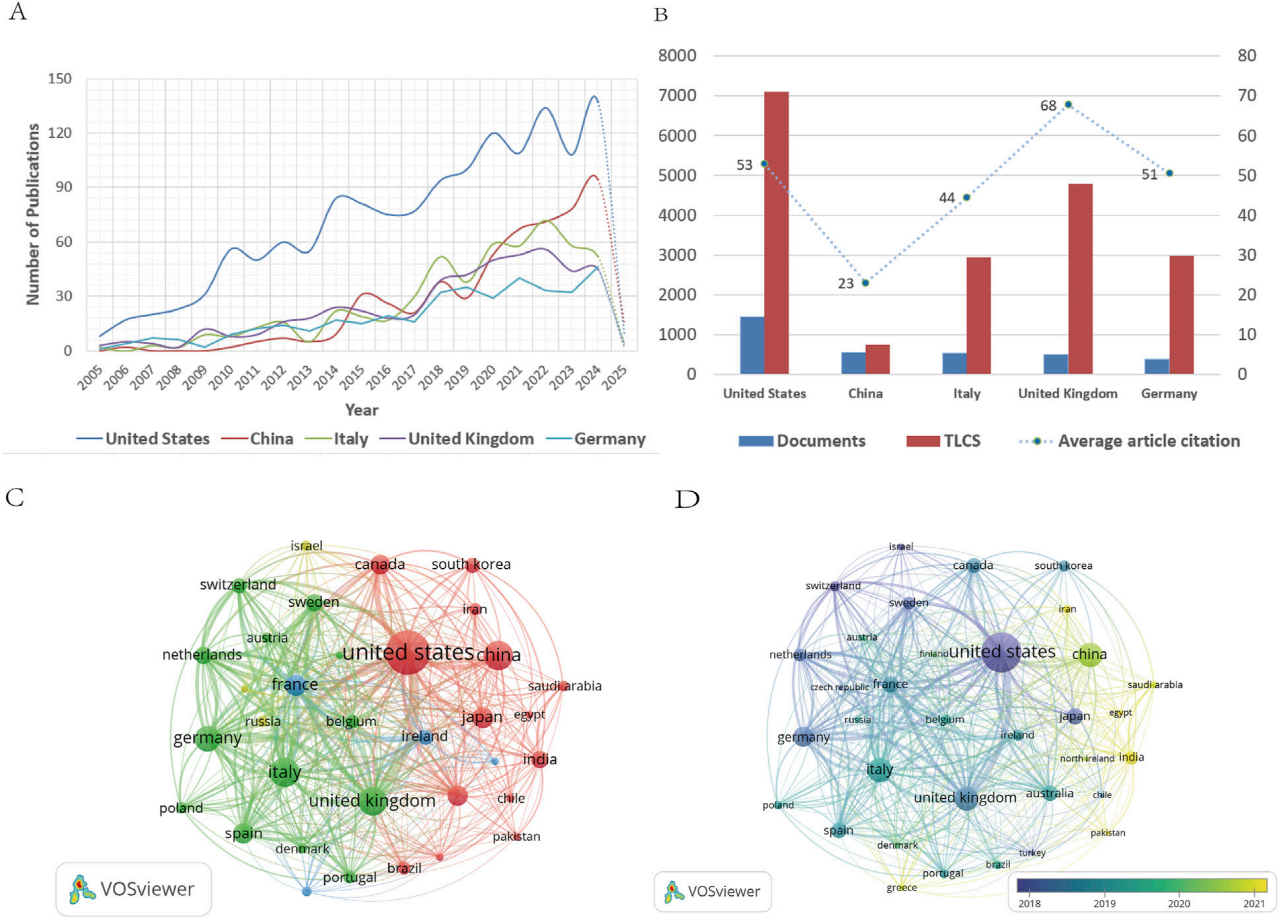
**(A)** Annual publication trends of the top five countries by publication volume. **(B)** Publication volume, TLCS, and average article citation of the top five countries by publication volume. **(C)** Country network visualization diagram. **(D)** Country overlay visualization diagram.

Country-specific developmental trajectories are illustrated in Figures 3A–D. Figure 3A highlights marked disparities among nations. The United States maintained dominance, with annual publications increasing from 8 (2005) to 137 (2024), a 17-fold growth, and entered a stable growth phase post-2019 (annual publications >100). China exhibited delayed but explosive growth, with minimal output from 2005 to 2009 followed by rapid escalation post-2010. Italy, the United Kingdom, and Germany experienced peak growth during 2021–2022, followed by moderated expansion. Figure 3B visualizes the publication volume, TLCS, and citation rates of the top five countries, reflecting uneven development across the field. The United States exemplified a “high-volume, moderate-to-high impact” profile, while the United Kingdom demonstrated “moderate-volume, high impact.” In contrast, China remained in a “moderate-volume, low impact” state. Although China’s late entry partially explains its lower TLCS due to delayed citation accumulation, efforts to enhance research quality are imperative.

VOSviewer’s network visualization (Figure 3C) depicts national contributions, where node size corresponds to publication volume and connection thickness indicates collaboration intensity. (Wang B. et al., 2024). The United States emerged as the central hub, while China, Italy, the United Kingdom, Germany, and Japan played pivotal roles in advancing the field. Overlay visualization (Figure 3D) uses color gradients to denote temporal activity. Deep blue nodes (the United States, United Kingdom, Italy, Germany) represent early-active nations, whereas yellow nodes (China, India, Iran, Saudi Arabia, Egypt, North Ireland, Pakistan, Greece) signify emerging contributors in recent years.

### 3.3 Institutional analysis

**FIGURE 4:**
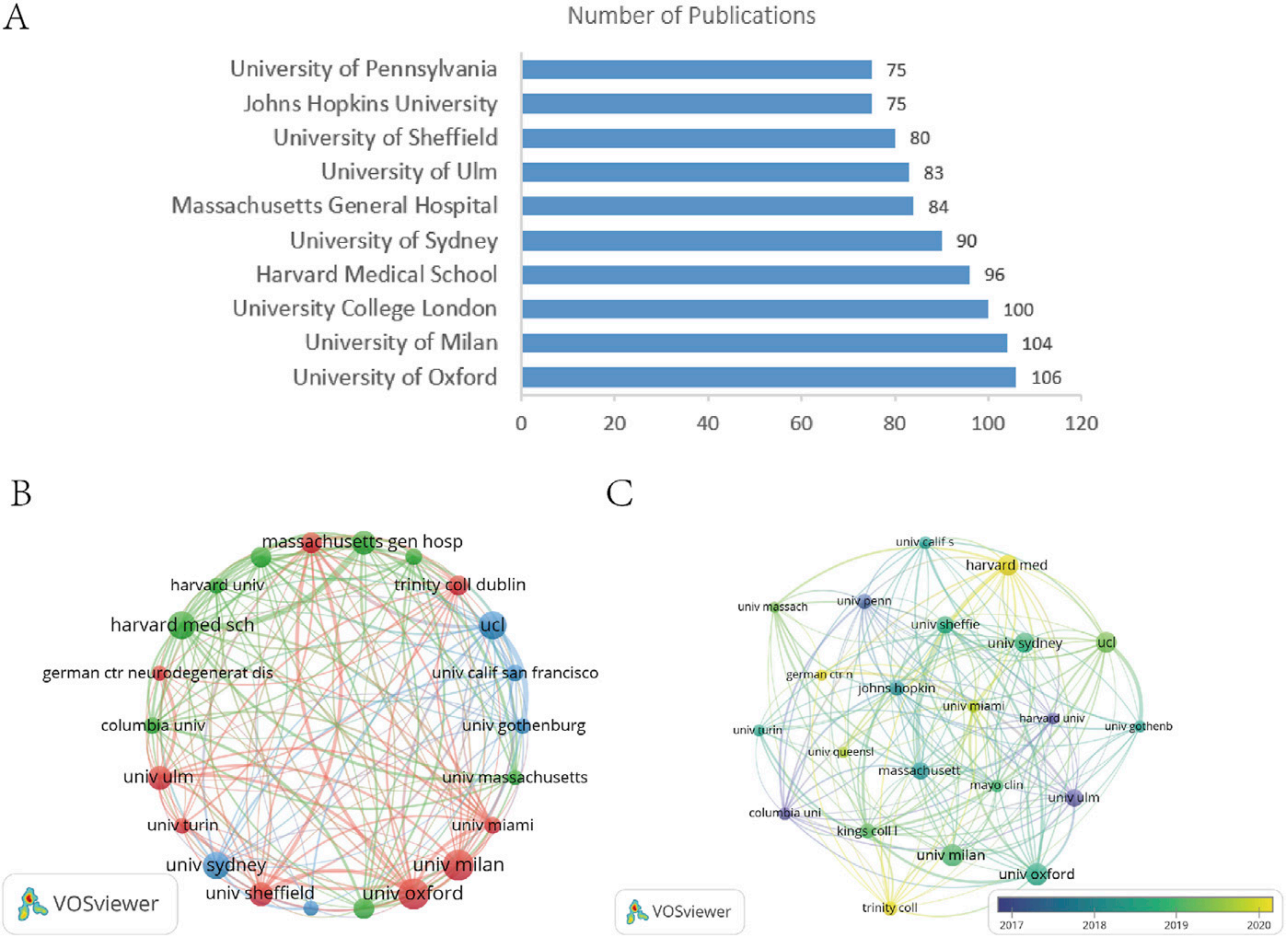
**(A)** Publication volume of the top 10 institutions by publication volume. **(B)** Institutional collaboration network visualization diagram. **(C)** Institutional overlay visualization diagram.

A total of 4,845 institutions participated in ALS-related research. Figure 4A lists the top 10 institutions by publication volume, revealing minimal disparities in output. The University of Oxford led with 106 publications, while the University of Pennsylvania and Johns Hopkins University ranked at the lower end with 75 publications each, indicating intense competition among institutions and the absence of a single dominant entity. Figure 4B presents a network visualization of 22 institutions with over 50 publications. Notably, the connection between Harvard University and Massachusetts General Hospital (MGH) was significantly thicker than others, underscoring their close collaboration. This partnership between a leading academic institution and a premier clinical center integrates basic and clinical research, driving scientific translation, technological breakthroughs, and substantial progress in the field. Figure 4C, an overlay visualization, highlights recent activity (around 2020) at institutions such as Harvard Medical School, the German Center for Neurodegenerative Diseases, the University of Miami, the University of Queensland, and Trinity College. Among the top five institutions by publication volume (Table 2), the University of Oxford demonstrated absolute dominance in both TLCS (17.66 per article) and TGCS, reflecting its core position in ALS research. University College London achieved a remarkable TGCS of 7,638 (76.38 per article), signifying its extensive influence in international interdisciplinary fields.

**TABLE 2:** Top five institutions with the most publications.

Lank	Institution	Country	Documents	Percent (%)	TLCS	TGCS
1	University of Oxford	United Kingdom	106	2.49	1872	6,078
2	University of Milan	Italy	104	2.45	832	4,680
3	University College London	United Kingdom	100	2.35	651	7,638
4	Harvard Medical School	United States	96	2.26	583	3,953
5	University of Sydney	Australia	90	2.12	558	3,411

### 3.4 Author analysis

**FIGURE 5:**
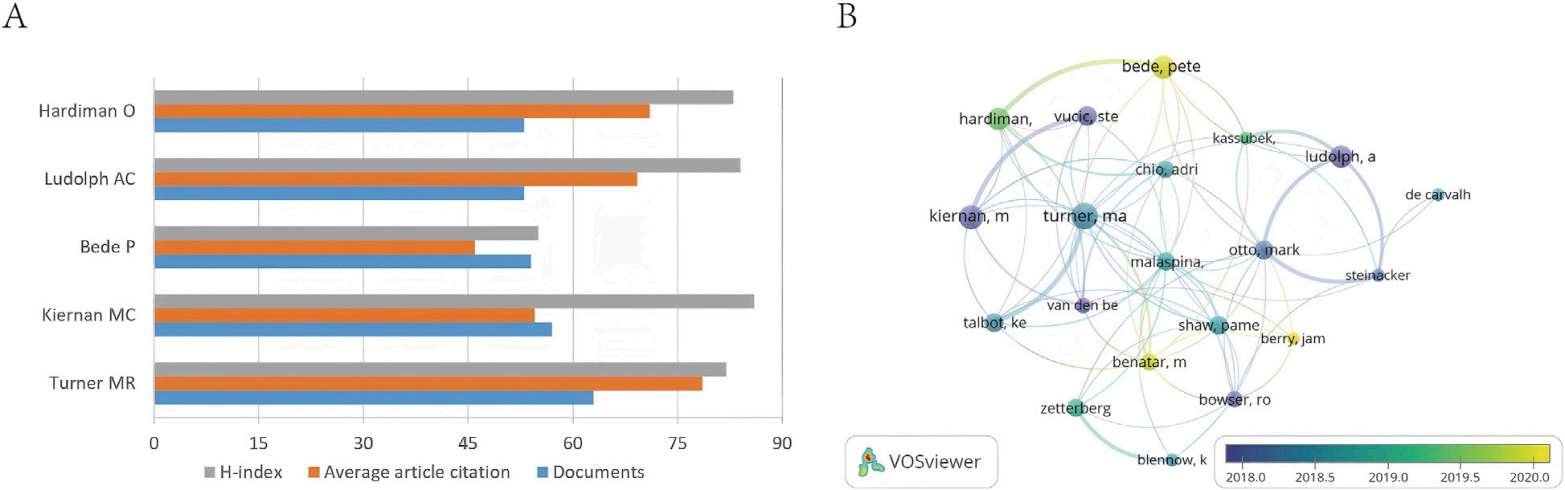
**(A)** Publication volume and average article citation of the top five authors by publication volume. **(B)** Author overlay visualization diagram.

A total of 20,168 authors contributed to ALS-related research. The top five authors by publication volume are summarized in Table 3. Turner MR emerged as the most prolific researcher, leading with 63 publications (2.78% of total output), an H-index of 82, and an average citation rate of 79 per article. His most globally cited work, titled “Amyotrophic Lateral Sclerosis” published in The Lancet (TGCS = 1,718), proposed the potential of the SOD1 gene as a presymptomatic biomarker and evaluated therapeutic strategies, including riluzole, symptomatic supportive medications (muscle relaxants, anticonvulsants, anticholinergic antidepressants, analgesics), and investigational agents such as insulin-like growth factor-1, lithium carbonate, minocycline, and stem-cell therapy. Turner MR’s TLCS (1,624) and TGCS (4,950) far exceeded those of the second-ranked author, Kiernan MC (TLCS = 695, TGCS = 3,107), solidifying his authority and international influence in the field. The top five authors collectively accounted for 6.59% of total publications, with their output ranging narrowly from 53 to 63 articles (Figure 5A), reflecting intense competition among leading researchers. Network visualization (Figure 5B) revealed robust collaborative networks among authors, with a surge of active contributors around 2020, including Bede Pete, Benatar M, and Berry Jam.

**TABLE 3:** Top five authors with the most publications.

Lank	Author	Documents	Percent (%)	TLCS	TGCS	Average article citation	H-index
1	Turner MR	63	2.78	1,624	4,950	79	82
2	Kiernan MC	57	2.62	695	3,107	55	86
3	Bede P	54	2.4	526	2,481	46	55
4	Ludolph AC	53	1.96	1,385	3,668	69	84
5	Hardiman O	53	1.8	680	3,762	71	83

### 3.5 Journal analysis

**FIGURE 6:**
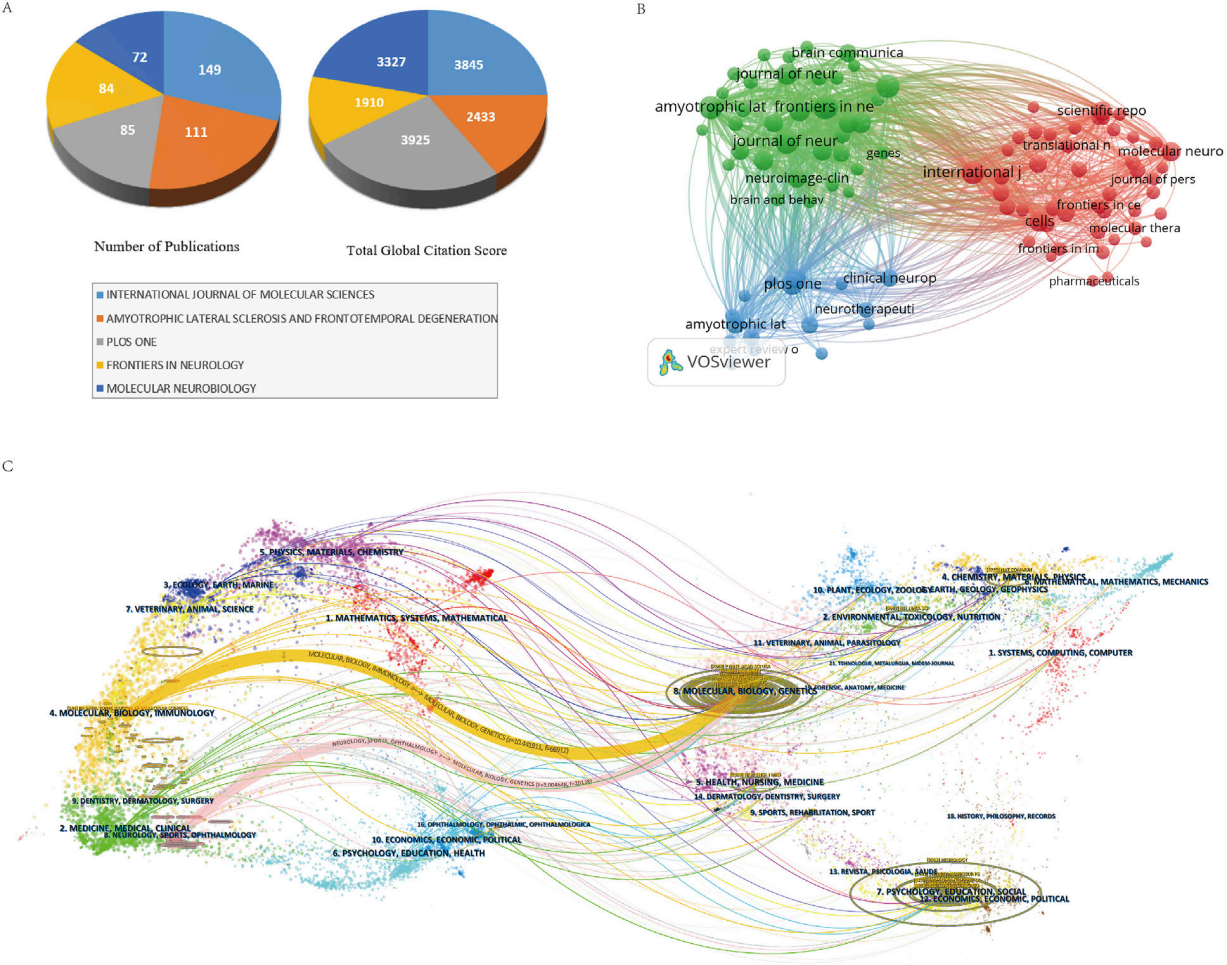
**(A)** Publication volume and TGCS of the top five journals by publication volume. **(B)** Journal collaboration network visualization diagram. **(C)** Journal double diagram overlay diagram.

The 4,250 publications were disseminated across 1,026 journals, with the top 10 journals by publication volume detailed in Table 4. The most prolific journals included “International Journal of Molecular Sciences” (N = 149, TGCS = 3,845), “Amyotrophic Lateral Sclerosis and Frontotemporal Degeneration” (N = 111, TGCS = 2,433), and “PLOS ONE” (N = 85, TGCS = 3,925), all of which significantly advanced ALS research. While “Amyotrophic Lateral Sclerosis” ranked second in publication volume (N = 111), its average citation rate (21.9 citations/article) and impact factor (2.5) lagged behind multidisciplinary journals such as “PLOS ONE” (46.2 citations/article), likely reflecting its specialized focus and narrower readership. Figure 6A identifies “PLOS ONE” (46 citations/article) and “Molecular Neurobiology” (46.2 citations/article) as high-impact journals within the field.

**TABLE 4:** Top 10 journals by publication volume.

Lank	Journal	Total publications	Total citations	Impact factor	SJR	H-index
1	International journal of molecular sciences	149	3,845	4.9	1.179	269
2	Amyotrophic lateral sclerosis and frontotemporal degeneration	111	2,433	2.5	1.066	85
3	Plos one	85	3,925	2.9	0.839	435
4	Frontiers in neurology	84	1910	2.7	0.966	105
5	Molecular neurobiology	72	3,327	4.6	1.339	140
6	Frontiers in neuroscience	69	1731	3.2	0.787	157
7	Muscle and nerve	67	1,547	2.8	0.964	167
8	Scientific reports	58	1,464	3.8	0.9	315
9	Cells	51	965	5.1	1.547	129
10	European journal of neurology	49	1,326	4.5	1.56	145

Figure 6B delineates three major journal clusters. The “green cluster”, encompassing journals like “Amyotrophic Lateral Sclerosis and Frontotemporal Degeneration”, “Frontiers in Neuroscience”, and “Genes”, primarily addresses neuroscience, clinical neurology, and genetics. The “blue cluster”, represented by “PLOS ONE”, “Clinical Neurophysiology”, and “Neurotherapeutics”, focuses on clinical neurophysiology and therapeutic interventions. The “red cluster”, dominated by “Molecular Neurobiology”, “International Journal of Molecular Sciences”, and “Cells”, centers on molecular biology, translational medicine, cellular neuroscience, immunology, and drug development.

To further investigate interdisciplinary knowledge flow, a dual-map overlay (Figure 6C) was constructed by superimposing citing journals (left) and cited journals (right). Two primary citation paths emerged:1. The first path, “Molecular Biology/Genetics → Molecular Biology/Immunology” (Z = 10.44, F = 66,912), exhibited high statistical significance (Z > 3) and substantial activity (F = 66,912), underscoring robust interdisciplinary synergy between these fields in driving ALS research.2. The second path, “Molecular Biology/Genetics → Neurology/Sports Medicine/Ophthalmology” (Z = 3.00, F = 20,128), though less prominent, demonstrated contributions from neurology, sports medicine, and ophthalmology to molecular biology and genetics, providing directional insights for ALS biomarker and therapy development. Both pathways converge on molecular biology and genetics, reaffirming their centrality in ALS research. These findings highlight the critical role of interdisciplinary integration, where diverse fields—ranging from molecular biology and immunology to clinical sciences—collaboratively propel advancements in understanding ALS pathogenesis and therapeutic innovation.

### 3.6 Co-citation analysis

Analysis of highly cited literature provides critical insights into the core research themes of the field. Table 5 summarizes the top 10 most frequently cited publications (2010–2020), which collectively explore the pathogenesis of ALS and lay the scientific foundation for identifying novel therapeutic targets, biomarkers, and drugs (Sweeney et al., 2018; Kim and Choi, 2015; Wardyn et al., 2015; Khalil et al., 2024; Kwon and Koh, 2020; Kim and Choi, 2010; Chiò et al., 2009; Mackenzie et al., 2010; Oakes and Papa, 2015; Ash et al., 2013). The most cited study, “Pathological Roles of MAPK Signaling Pathways in Human Diseases” by Kim et al. (2010) in “Biochimica et Biophysica Acta (BBA) - Molecular Basis of Disease”, elucidated how mutations in key genes such as SOD1, TDP-43, and FUS activate the JNK/p38 signaling pathways, leading to motor neuron damage through mechanisms involving neuroinflammation, oxidative stress, and cytoskeletal abnormalities. The study also highlighted the potential of targeting JNK/p38 pathways and their inhibitors as biomarkers and therapeutic strategies for ALS (Kim and Choi, 2010).

**TABLE 5:** Top 10 publications with the most citations.

Lank	Title	Journal	First author	Year	Total citations
1	Blood-brain barrier breakdown in Alzheimer disease and other neurodegenerative disorders	Nature reviews neurology	Sweeney, MD	2018	1871
2	Pathological roles of MAPK signaling pathways in human diseases	Biochimica et biophysica acta-molecular basis of disease	Kim, EK	2010	1863
3	Neurofilaments as biomarkers in neurological disorders	Nature reviews neurology	Khalil M	2018	1,308
4	Neuroinflammation in neurodegenerative disorders: the roles of microglia and astrocytes	Translational neurodegeneration	Kwon HS	2020	1,305
5	The Role of Endoplasmic Reticulum Stress in Human Pathology	Annual review of pathology	Oakes SA	2015	1,077
6	Unconventional Translation of C9ORF72 GGGGCC Expansion Generates Insoluble Polypeptides Specific to c9FTD/ALS.	Neuron	Ash PEA	2013	870
7	Dissecting molecular cross-talk between Nrf2 and NF-κB response pathways	Biochemical society transactions	Wardyn JD	2015	863
8	Compromised MAPK signaling in human diseases: an update	Archives of toxicology	Kim EK	2015	824
9	Prognostic factors in ALS: A critical review	Amyotrophic lateral sclerosis	Chiò A	2009	786
10	TDP-43 and FUS in amyotrophic lateral sclerosis and frontotemporal dementia	Lancet neurology	Mackenzie IRA	2010	715

**FIGURE 7:**
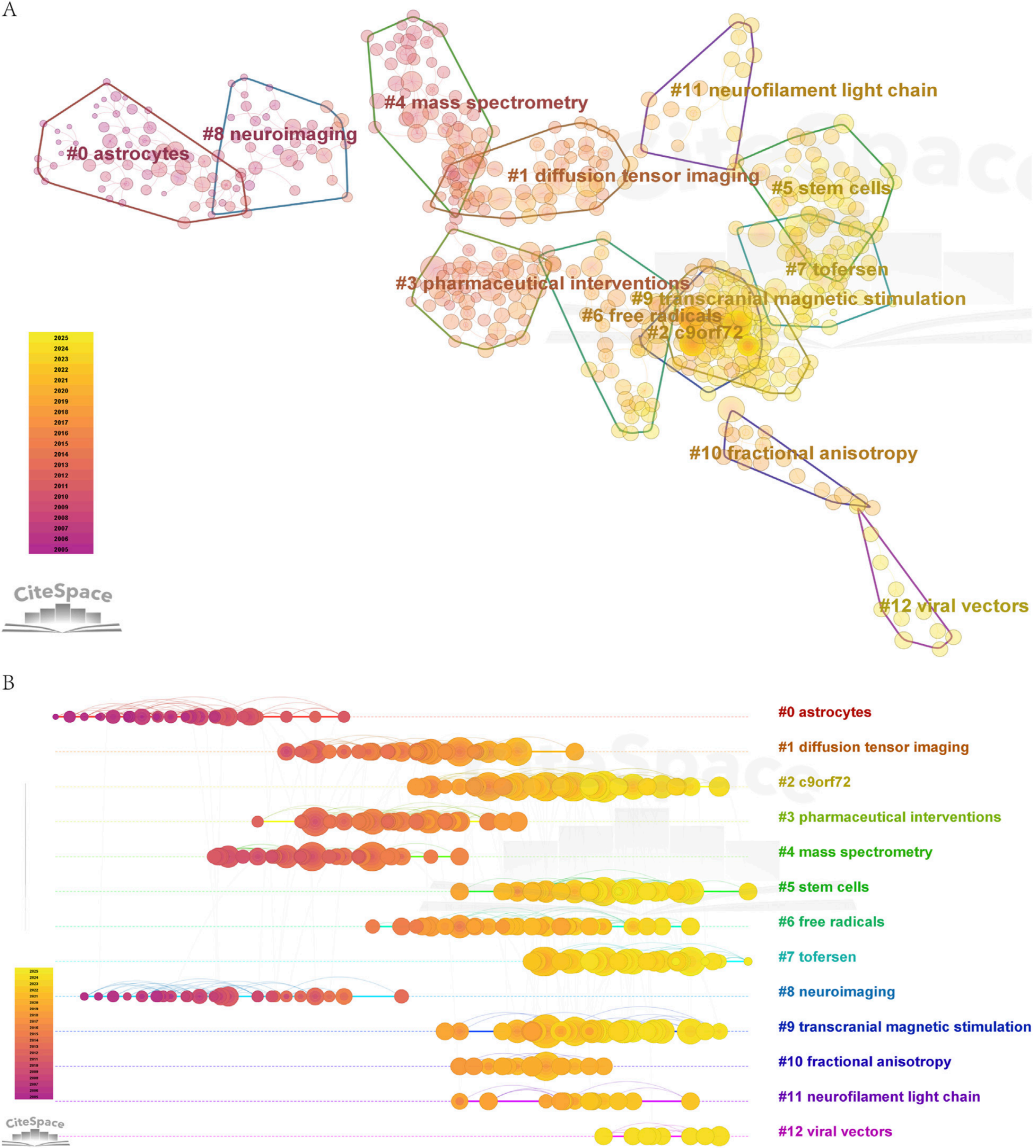
**(A)** Clustering diagram of co-cited documents. **(B)** Time evolution diagram of clustering of co-cited documents.

Clustering analysis of co-cited literature (Figure 7A) identified 13 thematic clusters: “#0 astrocytes, #1 diffusion tensor imaging, #2 c9orf72, #3 pharmaceutical interventions, #4 mass spectrometry, #5 stem cells, #6 free radicals, #7 tofersen, #8 neuroimaging, #9 transcranial magnetic stimulation, #10 fractional anisotropy, #11 neurofilament light chain, #12 viral vectors”. Five clusters—“diffusion tensor imaging, mass spectrometry neuroimaging, fractional anisotropy, neurofilament light chain”—were closely associated with biomarkers and diagnostic innovations. Neurofilament light chain (NfL), a highly sensitive biomarker for monitoring ALS progression, has remained a central research focus. Imaging technologies such as diffusion tensor imaging, mass spectrometry, and fractional anisotropy have advanced early diagnosis and subtype classification (Du et al., 2024). Six clusters—“c9orf72, pharmaceutical interventions, stem cells, tofersen, transcranial magnetic stimulation, viral vectors”—focused on therapeutic strategies. The “C9orf72” hexanucleotide repeat expansion, a major genetic factor in familial ALS, is linked to TDP-43 protein aggregation and dipeptide repeat protein (DPR) toxicity. Researchers are developing therapies targeting C9orf72, including antisense oligonucleotides (ASOs), RNA translation inhibitors, and gene therapies, though most remain in early clinical trials (Cook et al., 2020; Maharjan and Saxena, 2020). For example, “Tofersen”, an ASO targeting SOD1 mutations, is administered via intrathecal injection to suppress SOD1 mRNA translation, reducing SOD1 and NfL levels in cerebrospinal fluid. However, challenges include limited clinical efficacy, absence of Phase IV trials, high treatment costs, and the fact that SOD1 mutations account for only 2% of ALS cases (Miller TM. et al., 2022). Viral vectors serve as gene delivery tools for therapeutic and mechanistic studies, while stem cell therapy aims to replace damaged neurons and provide neurotrophic support (Giacomelli et al., 2022; Molecular mechanisms of amyotrophic lateral). Transcranial magnetic stimulation (TMS), used diagnostically to assess neuronal function and therapeutically to modulate cortical excitability, faces limitations such as restricted penetration depth, unclear mechanisms, and insufficient clinical validation (Ni and Chen, 2015). Two clusters—“astrocytes” and “free radicals”—highlighted pathological mechanisms. Astrocytes, traditionally viewed as neuronal support cells, are now recognized as key drivers of neuroinflammation, glutamate excitotoxicity, and mitochondrial dysfunction in ALS. Their dysregulation contributes to motor neuron degeneration, positioning them as critical therapeutic targets. Oxidative stress mediated by free radicals further exacerbates disease progression.

Timeline visualization (Figure 7) revealed evolving research hotspots, including “C9orf72” mutations, “Tofersen”, viral vectors, stem cell therapies, transcranial magnetic stimulation, and “neurofilament light chain” biomarkers. These clusters map the temporal and thematic distribution of ALS research, guiding future investigations into therapeutic and biomarker development.

### 3.7 Keyword analysis

**FIGURE 8:**
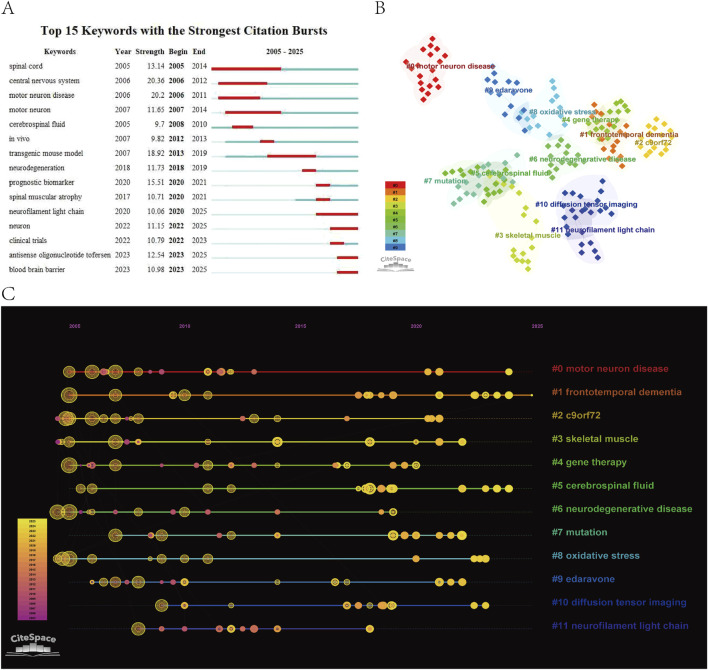
**(A)** Keyword burst diagram. **(B)** Keyword clustering diagram. **(C)** Keyword clustering time evolution diagram.

Keywords, extracted from titles, abstracts, and full texts of publications, are representative terms or phrases that succinctly capture the core themes and research foci of the literature. Figure 8A displays the top 15 keywords with the highest burst strength: “spinal cord, central nervous system, motor neuron disease, motor neuron, cerebrospinal fluid, *in vivo*, transgenic mouse model, neurodegeneration, prognostic biomarker, spinal muscular atrophy, neurofilament light chain, neuron, clinical trials, antisense oligonucleotide tofersen, blood-brain barrier”. Among these, “neurofilament light chain, neuron, antisense oligonucleotide tofersen”, and “blood-brain barrier” emerged as recent hotspots, highlighting the current focus on biomarkers related to neurofilament light chain, antisense oligonucleotide (ASO) therapies, and strategies to overcome the blood-brain barrier (BBB) for targeted drug delivery. The BBB, a specialized physiological barrier composed of brain microvascular endothelial cells, basement membranes, astrocytes, and pericytes, protects the brain from harmful substances while maintaining cerebral homeostasis. In some ALS patients, BBB integrity is compromised, allowing inflammatory cytokines and toxins to infiltrate the brain, exacerbating neuroinflammation and neuronal damage. Conversely, the BBB’s restrictive nature poses significant challenges for delivering large-molecule drugs and gene therapies to the central nervous system (CNS) (Md et al., 2019).

Keyword clustering aggregates terms with semantic or contextual similarities, offering insights into major research themes and hotspots. Figure 8B identifies 12 clusters: “#0 motor neuron disease, #1 frontotemporal dementia, #2 c9orf72, #3 skeletal muscle, #4 gene therapy, #5 cerebrospinal fluid, #6 neurodegenerative disease, #7 mutation, #8 oxidative stress, #9 edaravone, #10 diffusion tensor imaging, #11 neurofilament light chain”. Frontotemporal dementia (FTD), characterized by atrophy of the frontal and temporal lobes, co-occurs with ALS in up to 15% of patients. This overlap in clinical manifestations (executive dysfunction, language decline) and pathological mechanisms (TDP-43 protein aggregation, C9orf72 mutations, neuroinflammation, and glial activation) has reshaped the understanding of ALS as part of a “motor-cognitive continuum” within neurodegenerative diseases, driving innovations in therapeutic development (Ling et al., 2013). Skeletal muscle, the direct effector of motor neurons, undergoes denervation, fiber-type shifts, and mitochondrial dysfunction in ALS, yet biomarkers and therapeutic targets in skeletal muscle remain underexplored (Shefner et al., 2023). Oxidative stress, mediated by dysregulated astrocytes, microglia, and neurons, plays a critical role in ALS pathogenesis. Mutations in the antioxidant enzyme SOD1, associated with oxidative stress, account for approximately 20% of familial ALS cases. Edaravone, a free radical scavenger, is one of only two FDA-approved drugs for ALS. While its mechanism is well-defined and safety profile favorable, its modest efficacy (2-3 points improvement on the ALS Functional Rating Scale-Revised) and uncertain long-term survival benefits remain contentious (Abe et al., 2017). ALS pathogenesis is tightly linked to genetic mutations, with variations in genes such as “SOD1” and “SQSTM1” influencing age of onset, anatomical involvement (lower limb predominance), and phenotypic heterogeneity. Gene therapy, encompassing antisense oligonucleotides (ASOs), RNA interference (RNAi), and CRISPR/Cas9-based editing, represents a promising Frontier (Amado and Davidson, 2021). Cerebrospinal fluid (CSF), a critical medium for CNS homeostasis, harbors ALS biomarkers such as neurofilament proteins, SOD1, and tau. Beyond biomarker detection, CSF serves as a conduit for bypassing the BBB in drug delivery, enhancing therapeutic potential (Dreger et al., 2022).

Timeline visualization of keyword clusters (Figure 8C) underscores the rising prominence of FTD and CSF studies, reflecting a paradigm shift in ALS research from a “pure motor neuron disease” to a “motor-cognitive spectrum disorder.” This evolution highlights the integration of biomarker-driven approaches to bridge mechanistic exploration and clinical translation, transcending traditional disease boundaries and fostering interdisciplinary advancements.

## 4 Discussion

### 4.1 Precision breakthroughs in drug development and the caution against the dilution effect

Over the period from 2005 to 2024, global ALS research output surged 22-fold, with an average annual growth rate of 16.5%, accelerating to 19.8% post-2014. These two decades witnessed groundbreaking discoveries in clinical gene therapy, genetics, pathology, biomarkers, and imaging, with novel gene therapy paradigms now being applied to neurodegenerative diseases, including ALS. Researchers have elucidated the pathogenic mechanisms of “SOD1” and “C9orf72” mutations. Mutations in “SOD1” (superoxide dismutase 1), accounting for 10%–20% of familial ALS cases, impair copper and zinc binding due to structural rearrangements in variants such as H46 R and G85R, destabilizing fibril conformations and triggering ferroptosis (Wang LQ. et al., 2024). Additionally, “SOD1” mutants accumulate in mitochondrial intermembrane spaces, disrupting electron transport chain activity, impairing mitochondrial function, and ultimately leading to metabolic failure and neuronal death. The “C9orf72” (G4C2) repeat expansion, the most common genetic cause of familial ALS (20%–50% of cases), suppresses C9orf72 transcription, reducing protein expression and driving neuronal dysfunction, mitochondrial defects, immune dysregulation, and DNA damage. Leveraging these insights, antisense oligonucleotide (ASO) therapies targeting “SOD1” and “C9orf72” have advanced to clinical trials (Van Daele et al., 2024). For instance, “Tofersen” (targeting “SOD1” mRNA) underwent intensive Phase I/II trials from 2016 to 2019, inspired by post-2014 mechanistic breakthroughs. In October 2024, Tofersen became the first ALS gene therapy approved in China. However, annual publication trends reveal a “dilution effect,” with declining TLCS post-2019, reflecting a proliferation of low-quality, repetitive studies—such as generalized oxidative stress analyses—that lack clinical translation. For example, 2019–2024 saw a surge in “*in vitro*” studies on “free radical scavengers” yet few progressed to clinical validation. Future efforts must prioritize multi-omics integration (combined single-cell transcriptomics and proteomics) and organoid models to enhance target screening efficiency, mitigate resource waste, and ensure precision in therapeutic innovation.

### 4.2 Translational bottlenecks and clinical challenges in gene and stem cell therapies

The co-citation analysis highlights “Tofersen” (Cluster #7) as a prominent keyword in recent years. However, Phase III clinical trials revealed that while “Tofersen” reduces SOD1 protein and neurofilament light chain (NfL) levels in cerebrospinal fluid (CSF), it failed to significantly improve ALS Functional Rating Scale-Revised (ALSFRS-R) scores (Hamad et al., 2025). This discrepancy underscores the limitations of targeting “SOD1”, which accounts for only 2% of ALS cases, with mutation-specific responses (A4V vs D90A) further complicating therapeutic efficacy. There is an urgent need to identify pan-mutation therapeutic targets and develop corresponding therapies. Additionally, the insensitivity of ALSFRS-R to early-stage disease progression and the exclusion of biomarkers like NfL from primary endpoints hinder objective efficacy evaluation. Adaptive clinical trial designs incorporating dynamic NfL monitoring are now underway to enhance assessment accuracy.

Toxic dipeptide repeat proteins (DPRs) driven by “C9orf72” mutations represent a current research focus. Antisense oligonucleotides (ASOs) such as IONIS-C9Rx and CRISPR-Cas9-mediated repeat deletion have shown efficacy in animal models, yet clinical translation faces challenges: (1) C9orf72 is widely expressed in microglia and astrocytes, raising concerns about systemic gene editing disrupting immune function; (2) 90% of ALS cases are sporadic, with mechanisms diverging from C9orf72-linked pathology, necessitating broad-spectrum therapies.

Stem cell therapy has emerged as another transformative approach, leveraging stem cells’ capacity to differentiate into neurons, delay disease progression, and improve symptoms. Recent clinical studies demonstrate its potential to slow ALS advancement and enhance quality of life. For instance, a 2020 study by Pomeranian Medical University in “Cells” revealed that repeated intrathecal administration of autologous bone marrow-derived lineage-negative stem/progenitor cells modulates immune pathways and may halt neurodegeneration (Baumert et al., 2020). A 2021 Phase III trial led by Massachusetts General Hospital (Muscle & Nerve) reported that mesenchymal stem cell-neurotrophic factor (MSC-NTF) therapy was well-tolerated, with 33% of MSC-NTF recipients *versus* 28% of placebo recipients meeting clinical response criteria at 28 weeks. MSC-NTF treatment significantly improved CSF biomarkers of neuroinflammation, neurodegeneration, and neurotrophic support (Cudkowicz et al., 2022). However, critical barriers persist: (1) immune rejection of transplanted cells; (2) tumorigenic risks from undifferentiated stem cells; (3) poor survival and integration of stem cells in ALS’s toxic pathological milieu; and (4) technical challenges in targeted delivery to the nervous system *via* methods like intrathecal injection or spinal transplantation, which carry complication risks. Future strategies should integrate biomaterials (hydrogel-based slow-release carriers) and genetic engineering (neurotrophic factor overexpression in MSCs) to enhance therapeutic precision and efficacy.

### 4.3 The emerging potential of blood-brain barrier penetration and skeletal muscle targeting in ALS

Keyword burst analysis identifies “blood-brain barrier penetration” (Burst Strength = 10.98) and “skeletal muscle targeting” (Cluster #3) as emerging research directions in ALS. The blood-brain barrier (BBB), a major obstacle to central nervous system (CNS) drug delivery, limits brain concentrations of traditional drugs like edaravone to less than 1%. Novel delivery systems, however, have significantly enhanced CNS drug levels in preclinical models. Transferrin receptor (TfR) antibody-mediated exosome-based drug delivery, leveraging exosomes’ innate BBB-penetrating capacity and low immunogenicity, is a current focus. Studies demonstrate that engineered exosomes loaded with siRNA can target TfR to reduce β-amyloid deposition in Alzheimer’s mouse models. In ALS, TfR antibody-modified exosomes carrying SOD1 antisense oligonucleotides (ASOs) achieved 15% drug concentration in cerebrospinal fluid and delayed motor neuron degeneration by 30% in SOD1-G93A transgenic mice (Wang et al., 2023; Yang and Zhang, 2022). Focused ultrasound (FUS) combined with microbubbles, a technique validated for BBB opening in Alzheimer’s trials, enhances ASO delivery efficiency in ALS (Gao et al., 2024). FUS selectively opens the BBB in hippocampal regions, increasing brain concentrations of antibody drugs (aducanumab) fivefold. In ALS models, FUS combined with intrathecal Tofersen administration improved ASO distribution in spinal motor neuron regions fourfold and significantly restored motor function in SOD1 mice. However, BBB opening risks localized neuroinflammation (Cheng et al., 2024), necessitating optimized ultrasound parameters to balance efficacy and safety. (Ultrasound-induced blood; Ediriweera et al., 2025).

While traditional ALS research emphasizes motor neuron degeneration, recent evidence highlights skeletal muscle pathology as an early and independent contributor to disease progression. For instance, muscle-specific kinase (MuSK) activators like agrin-mimetic peptides delay atrophy in SOD1 mice, though human trials are hindered by poor oral bioavailability, prompting exploration of subcutaneous sustained-release formulations or muscle-targeted gene therapies. Skeletal muscle mitochondrial dysfunction drives reactive oxygen species (ROS) accumulation, and Viral vectors serve as gene delivery tools for therapeutic and mechanistic studies, while stem cell therapy aims to replace damaged neurons and provide neurotrophic support (Xue et al., 2024). antioxidant combination therapies—edaravone (a free radical scavenger) with coenzyme Q10 (a mitochondrial electron transport chain cofactor)—exhibit synergistic effects in preclinical models (Vanhauwaert et al., 2024; Chen et al., 2024). Furthermore, gene editing can also be applied to muscle tissue. For instance, CRISPR/Cas9 technology enables precise gene editing within skeletal muscle cells, allowing for the correction of disease-causing DNA mutations. This approach holds promise for addressing genetic defects directly at the muscular level, offering a potential therapeutic pathway for conditions linked to skeletal muscle dysfunction (Yang et al., 2016). Future interdisciplinary approaches integrating FUS-mediated BBB modulation with skeletal muscle-targeted therapies could enable dual “central-peripheral” interventions, addressing both CNS and peripheral pathology in ALS.

Interdisciplinary collaboration with computational neuroscience is crucial for overcoming current translational bottlenecks. For instance, machine learning algorithms can analyze multi-omics datasets (genomics, proteomics, neuroimaging) to identify novel biomarkers or predict treatment responses. Similarly, computational models of the blood-brain barrier dynamics can optimize focused ultrasound parameters (frequency, pulse duration) to maximize drug penetration while minimizing the risk of neuroinflammation. As with physiologically based pharmacokinetic (PBPK) modeling applied in oncology, it may similarly guide dosing regimens for antisense oligonucleotides or stem cell therapies.

### 4.4 Limitations and future directions

This study has several limitations. First, the literature search was restricted to English-language databases, potentially omitting therapeutic advancements reported in non-English publications. Moreover, despite our efforts to enrich the search terms and refine the search strategy, it cannot be denied that a very small number of relevant terms may have been overlooked. Second, burst detection analysis, influenced by citation lag, may not fully capture emerging technologies such as CRISPR gene editing, multi-layer graph attention neural networks (MLGANN), and bimodal neural networks. Future research should integrate multimodal data (clinical trial registries, single-cell transcriptomic databases) and develop AI-driven target prediction models to enhance discovery efficiency. Additionally, fostering global collaboration through initiatives like the International ALS Genomics Consortium and establishing comprehensive patient registries (the European ENROLL platform) will accelerate the translation of foundational discoveries into clinical applications. These efforts will bridge gaps between bench-to-bedside research and address unmet needs in ALS diagnosis, biomarker validation, and therapeutic innovation.

## 5 Conclusion

This study systematically uncovers the evolving trends and core challenges in ALS research. Global annual publications expanded at a rate of 16.5%, yet a significant “quantity-quality imbalance” persists: the United States dominates in output (34.07% of total publications), but its per-article impact (53 citations) lags behind the United Kingdom (68 citations). China, despite ranking second in publication volume (553 articles), achieved a Total Local Citation Score (TLCS) merely one-tenth of that of the U.S., highlighting the urgency of transitioning from “quantity-driven” to “quality-prioritized” research. Biomarker research remains central to bridging mechanistic insights and clinical translation. Neurofilament light chain (NfL) has emerged as a sensitive prognostic marker, yet its specificity across heterogeneous populations requires refinement. Imaging biomarkers (diffusion tensor imaging, fractional anisotropy) enable non-invasive subtyping but face standardization barriers. Future integration of multi-omics data (extracellular vesicle miRNAs, TDP-43 phosphorylation patterns) with AI-driven models could enhance diagnostic precision. Dynamic NfL monitoring may optimize ASO dosing (Tofersen), while CSF biomarkers (SOD1 levels) offer real-time feedback for gene therapy. Biomarker-guided stratified therapies (C9orf72 mutation-based interventions) are critical for personalized medicine. Therapeutically, antisense oligonucleotide (ASO) drugs effectively reduce SOD1 protein and neurofilament light chain (NfL) levels but fail to improve clinical endpoints due to target limitations and insufficient delivery efficiency. Stem cell therapies face bottlenecks such as off-target risks and immune responses. Emerging strategies show promise: transferrin receptor antibody-modified exosomes enhance intracranial drug concentrations, while focused ultrasound combined with microbubbles improves ASO distribution in spinal regions. Skeletal muscle, as an early intervention target, exhibits mitochondrial dysfunction preceding neuronal death. However, muscle-specific kinase (MuSK) activators are hindered by poor oral bioavailability, necessitating future exploration of muscle-targeted adeno-associated virus (AAV) gene therapies or CRISPR-based editing. The 2014 *Ice Bucket Challenge* catalyzed ASO development, underscoring the need for dedicated funding initiatives to support ALS therapeutic innovation, which would profoundly benefit patients. Moving forward, deepening interdisciplinary collaboration, optimizing resource allocation, and prioritizing technological breakthroughs will provide critical pathways for transformative advancements in ALS treatment paradigms.

## Data Availability

The original contributions presented in the study are included in the article/supplementary material, further inquiries can be directed to the corresponding author.
